# Performance evaluation of the NG-TEST CARBA 5 and Genobio K.N.I.V.O. detection K-Set lateral flow assays for the detection of carbapenemases

**DOI:** 10.1128/spectrum.00441-25

**Published:** 2025-07-18

**Authors:** Chloe N. Calica, Neda Domjacic, Jennifer Dacanay, Ethel C. de Carvalho Lopes, Mariel S. Mardoquio, Yan Chen, Ramzi Fattouh

**Affiliations:** 1 Bioinformatics and Computational Biology, University of Toronto 7938, Toronto, Ontario, Canada; 2 Division of Microbiology, Department of Laboratory Medicine, Unity Health Toronto 508783, Toronto, Ontario, Canada; 3 Department of Laboratory Medicine and Pathobiology, Temerty Faculty of Medicine, University of Toronto 233837, Toronto, Ontario, Canada; 4 Li Ka Shing Knowledge Institute, Unity Health Toronto 508783, Toronto, Ontario, Canada; Icahn School of Medicine at Mount Sinai, New York, New York, USA

**Keywords:** clinical microbiology, medical microbiology, bacteria, antibiotic resistance, carbapenemase, diagnostics

## Abstract

Infection with carbapenemase-producing organisms (CPOs) is associated with significant morbidity and mortality among hospitalized patients. Thus, the identification of CPOs is an important function of clinical microbiology laboratories. This study evaluates the performance of two lateral flow assays (LFAs), the NG-Test CARBA 5 (CARBA 5) and Genobio K.N.I.V.O. Detection K-Set (Genobio), for the detection of carbapenemases using well-characterized clinical and reference isolates of gram-negative bacterial species. Both LFAs are designed to detect KPC, NDM, VIM, IMP, and OXA-48-like carbapenemases from bacterial growth within 15 minutes. In total, 152 isolates were tested: 10 *Acinetobacter baumannii*, 12 *Pseudomonas aeruginosa*, and 130 from various *Enterobacterales*. Among these, 132 harbored a carbapenemase, and 20 were carbapenemase-negative. Additionally, LFAs were scored for background color and test indicator line intensity. For *Enterobacterales*, the CARBA 5 had an overall positive agreement of 100%, while Genobio had a positive agreement of 98.5% because of a failure to detect IMP in a *Klebsiella pneumoniae* isolate and false cross-reactivity for NDM and OXA-48 in a KPC-positive *Morganella morganii*. Both LFAs demonstrated a positive agreement of 100% among carbapenemase-producing *A. baumannii* and *P. aeruginosa* isolates. Neither LFA falsely detected carbapenemases among carbapenemase-negative isolates, nor was any positivity observed among isolates that carried an off-target carbapenemase. In terms of test indicator line intensity, both LFAs showed comparable performance; however, the Genobio LFA displayed a propensity for higher background signal. Overall, both LFAs demonstrated very good performance in detecting select carbapenemases and offered a fast turnaround time, straightforward interpretation, and minimal hands-on time.

**IMPORTANCE:**

This study contributes to the field by providing additional information on the performance of two commercial tests, known generally as lateral flow assays (LFA). These assays are used to identify a family of proteins, called carbapenemases, that cause antibiotic resistance and greatly limit the choices of antibiotics that can be used to treat infection. Our study evaluates the performance of two LFAs in a variety of bacteria, including some that are commonly studied (members of the *Enterobacterales*) as well as others where less test performance information is available (*Pseudomonas aeruginosa* and *Acinetobacter baumannii*). Our findings show that the LFAs provide a fast turnaround time and require minimal hands-on time in comparison to other tests. In addition, we provide information about practical factors that could negatively affect how the results are interpreted in busy clinical laboratories, such as the ease of “seeing” and interpreting the results.

## INTRODUCTION

The prevalence of carbapenemase-producing organisms (CPOs) and the incidence of infection with CPOs continue to rise in Canada and worldwide (1). Among the CPOs of greatest concern are several members of the *Enterobacterales, Acinetobacter baumannii*, and *Pseudomonas aeruginosa* (2). These organisms have the potential to transmit resistance via mobile genetic elements widely, and they often possess resistance mechanisms to multiple antibiotic classes, making infections exceptionally difficult to treat (2). For these reasons, detecting CPOs, particularly carbapenemase-producing *Enterobacterales*, at the institutional level is a cornerstone of infection prevention and control efforts. Moreover, carbapenemase type determination is valuable for the management of clinical infection with CPO as newer generations of antimicrobials display differential efficacy against carbapenemase types. Thus, rapid and accurate detection of carbapenemases is a pivotal role of front-line clinical microbiology laboratories.

The Clinical and Laboratory Standards Institute M100-Ed34 recommends phenotypic-based methods for detecting carbapenemase production among *Enterobacterales* and *P. aeruginosa*, such as the modified carbapenemase inactivation method (mCIM) and Carba NP. Both methods demonstrate good performance in detecting CPOs and can be relatively inexpensive; however, they are labor-intensive, can be challenging to interpret, require incubation on the order of hours, and for the Carba NP, involve the preparation of several reagents (3–7). In contrast, a commercially available lateral flow assay (LFA), the NG-Test CARBA 5 (CARBA 5), has displayed consistent high-level performance (sensitivity of 88%–100% and specificity of 96%–100%) (8–18). This assay detects the most prevalent carbapenemases (KPC, NDM, OXA-48-like, VIM, and IMP) in *Enterobacterales* and *P. aeruginosa*. Although data are limited, a second LFA, Genobio K.N.I.V.O. Detection K-Set (Genobio), has shown promising performance (sensitivity 96%–100% and specificity 93%–100%) (16, 18–20). Fewer studies have evaluated the performance of LFAs for the detection of carbapenemases in *P. aeruginosa* and *A. baumannii*. Although limited, investigations that have included these organisms show encouraging results for the detection of several carbapenemase types with the exception of IMP carbapenemases, where many variants are missed by LFAs (9, 11, 14, 16, 19).

This study aimed to evaluate the performance of the CARBA 5 and Genobio assays using well-characterized *Enterobacterales*, *P. aeruginosa, and A. baumannii* isolates. Additionally, the LFA test indicator line intensity and background signal were also evaluated, as these factors, to our knowledge, have not been previously described. These factors can cause uncertainty or even false-negative interpretations in the setting of a busy frontline clinical lab.

## MATERIALS AND METHODS

### Bacterial isolates

A total of 152 isolates were employed in this study. The majority of these isolates (*n* = 111) came from three panels of the Centers for Disease Control and Prevention (CDC) and U.S. Food and Drug Administration (FDA) Antibiotic Resistance (AR) Isolate Bank, namely, the *Enterobacterales* Carbapenem Breakpoint panel, the *Enterobacterales* Carbapenemase Diversity panel, and the Gram-Negative Carbapenemase Detection panel. The remaining 41 clinical isolates were obtained from clinical testing at St. Michael’s Hospital, Unity Health Toronto. These clinical isolates were sent to the provincial reference laboratory (Public Health Ontario Laboratories; PHOL), as part of routine protocols, for carbapenemase detection using a clinically validated real-time PCR test that targets KPC, NDM, GES, OXA-48-like, VIM, and IMP genes (21); sequencing-based characterization of these 41 isolates was not performed.

All isolates were recovered from frozen beads and were first inoculated onto Modified MacConkey with Carbapenem agar (Cat#: MP0409; Oxoid Thermo Fisher Scientific, Ontario, Canada), which was incubated overnight at 35°C. The following day, the growth was subcultured onto a standard MacConkey agar (MAC; Cat#: MP1312; Oxoid Thermo Fisher Scientific) and incubated as before. Isolated colonies that grew on the MAC plate were then selected for carbapenemase detection employing both commercial LFAs.

### NG-Test CARBA 5 (NG Biotech Laboratories, United States) and GENOBIO K.N.I.V.O (Gold Mountain River, China) lateral flow assays for carbapenemase detection

Isolates were tested in batches, typically in groups of 10–20, and run in parallel on both LFAs. Testing was performed according to the manufacturers’ instructions. A full 1 µL inoculation loop of bacteria from the MAC plate was mixed with five drops of extraction buffer and then vortexed for about 10 seconds before immediate use. For the CARBA 5 LFA, mucoid colonies were vortexed for 3 minutes and then incubated for 10 minutes at room temperature before testing. Subsequently, 100 µL of the mixture was dispensed into the cartridge, and results were read at 15 minutes of incubation. Both LFAs detect five types of carbapenemases, including KPC, NDM, VIM, IMP, and OXA-48-like carbapenemases. Additionally, background smearing and carbapenemase-type indicator line intensity were scored by visualization for each LFA test performed by the same operator. Background smearing intensity was scored as “none” (no background color), “cloudy weak” (partial smearing), or “cloudy strong” (intense smearing), depending on the extent of the cartridge background color. The intensity of the carbapenemase type indicator line was scored as “bright” (strong and uniform signal) or “dim” (faint signal).

### Precision study for LFAS

Three isolates were used to conduct the precision study: a KPC-3-producing *Enterobacter cloacae* complex, a VIM-1-producing *Klebsiella pneumoniae*, and a carbapenemase-negative *Klebsiella oxytoca*. Over 3 days, four different technologists performed the two LFA tests on the selected isolates, using two different kit lots.

### Statistical analysis

Concordance was calculated for CARBA 5 and Genobio, in comparison with the reference results from the CDC and FDA AR Isolate Bank and PHOL. The CARBA 5 assay produced one invalid result. This was excluded from the concordance calculation for the CARBA 5 assay.

## RESULTS

### Overview of gram-negative bacteria included in this study

In total, 152 well-characterized isolates were evaluated using the CARBA 5 and Genobio LFAs. These isolates include 10 *A. baumannii*, 12 *P. aeruginosa*, and 130 from multiple species within the order *Enterobacterales* (Table S1). Among the studied isolates, 132 were positive for carbapenemases (37 KPC, 36 NDM, 25 OXA-48, 13 VIM, 9 SME, 6 IMP, 2 NMC, 2 IMI, and 2 NDM/OXA-48-like) and 20 were carbapenemase-negative (Table S1).

### LFA performance for the detection of carbapenemases in *Enterobacterales*, *Pseudomonas aeruginosa,* and *Acinetobacter baumannii*

For *Enterobacterales*, both CARBA 5 and Genobio showed excellent ability to detect the five carbapenemases they target with an overall concordance of 100% and 98.5%, respectively (Table 1). The Genobio LFA failed to detect IMP-8 in a *K. pneumoniae* isolate; two attempts were made to test this isolate, and both were negative. In addition, one KPC-producing *Morganella morganii* isolate tested positive for KPC as expected; however, it was also found to be falsely positive for both NDM and OXA-48 by the Genobio test, which was observed on repeat testing (Fig. S1). The CARBA 5, but not the Genobio, LFA produced one invalid result from a mucoid non-carbapenemase producing *K. pneumoniae*, out of 50 isolates that were observed to display a mucoid colony morphology (Table 1 and data not shown). LFA concordance for *P. aeruginosa* and *A. baumannii* isolates was 100% for both assays (Table 1). Of note, a limited spectrum of carbapenemases was studied based on availability: NDM for *A. baumannii* and KPC, VIM, and IMP for *P. aeruginosa*.

**TABLE 1:** Performance of the NG-Test CARBA 5 (CARBA 5) and Genobio K.N.I.V.O. (Genobio) lateral flow assays (LFAs) for the detection of five types of carbapenemases^*g*^

	CP^*a*^	Genobio		CARBA 5	
		No.^*b*^	No. (%) concordant	No.^*b*^	No. (%) concordant
***A. baumannii***	NDM	4	4 (100)	4	4 (100)
	NEG^*c*^	6	6 (100)	6	6 (100)
	**Total**	**10**	**10** (**100**)	**10**	**10** (**100**)
***P. aeruginosa***	KPC	1	1 (100)	1	1 (100)
	VIM	5	5 (100)	5	5 (100)
	IMP	2	2 (100)	2	2 (100)
	NEG	4	4 (100)	4	4 (100)
	**Total**	**12**	**12** (**100**)	**12**	**12** (**100**)
***Enterobacterales***	KPC	36	35^*e*^ (97.2)	36	36 (100)
	NDM^*d*^	34	34 (100)	34	34 (100)
	OXA-48^*d*^	27	27 (100)	27	27 (100)
	VIM	8	8 (100)	8	8 (100)
	IMP	4	3 (75.0)	4	4 (100)
	NEG	23	23 (100)	22^*f*^	22 (100)
	**Total**	**132**	**130** (**98.5**)	**131^*f*^**	**131** (**100**)

^*a*^ CP, carbapenemase.

^*b*^ No., number of isolates tested.

^*c*^ NEG includes carbapenemase-negative *Enterobacterales*, *A. baumannii,* and *P. aeruginosa* isolates and isolates that produce a carbapenemase (IMI, NMC-A, and SME) that is not detected by either LFA.

^*d*^ Two isolates contained both NDM and OXA-48, so each carbapenemase was interpreted separately by type.

^*e*^ One KPC-producing *M. morganii* isolate tested positive for KPC and falsely positive for NDM and OXA-48, and was therefore considered discordant.

^*f*^ The CARBA 5 LFA produced one invalid result from a mucoid non-carbapenemase-producing *K. pneumoniae*, which was omitted from the concordance calculation.

^*g*^ Bold indicates the difference between the Cumulative data and granular data.

Neither LFA falsely detected carbapenemases in 11 *Enterobacterales*, 4 *P*. *aeruginosa*, and 6 *A*. *baumannii* isolates that were negative for carbapenemases, nor was any positivity observed among the 12 *Enterobacterales* isolates that carried carbapenemases other than the five types detected by the LFAs. Therefore, the negative concordance of both LFAs was found to be 100% (Table 1).

LFA precision was assessed by repeat testing of three isolates (two carbapenemase-positive and one carbapenemase-negative) on separate days, with differing operators, and two different lots of reagents. Both the CARBA 5 and Genobio LFAs demonstrated a 100% precision rate (data not shown).

### LFA background color and line intensity

**Fig 1:**
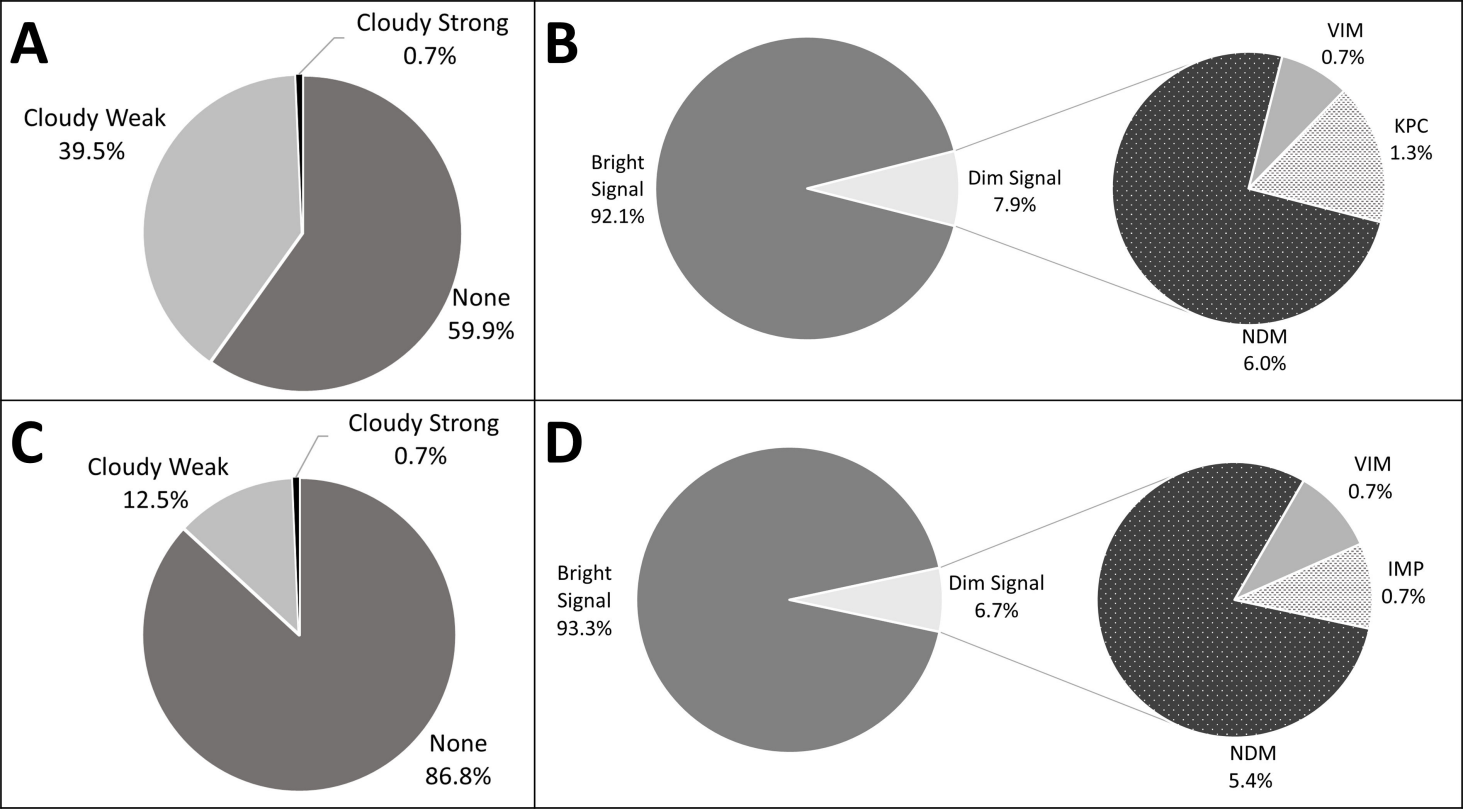
Qualitative evaluation of lateral flow assay (LFA) background smearing and test line intensity. (**A**) Background smearing intensity for the Genobio K.N.I.V.O. (**B**) Signal intensity of the carbapenemase type indicator line for the Genobio K.N.I.V.O. (**C**) Background smearing intensity for the NG-Test CARBA 5. (**D**) Signal intensity of the carbapenemase type indicator line for the NG-Test CARBA 5.

The presence of background smearing and carbapenemase-type indicator line intensity was also evaluated at various time points (15, 20, and 25 minutes of incubation). Background smearing intensity was scored as “none,” “cloudy weak,” or “cloudy strong,” depending on the extent of the test background color (Fig. S2). The CARBA 5 assay was more likely to produce a result without any background smearing (87% of tests performed) as compared to the Genobio assay, where only 59% of tests displayed no background smearing (Fig. 1). For those instances where background smearing was noted at the 15 minute read, they were predominantly “cloudy weak” in appearance.

The intensity of the carbapenemase type indicator line was also scored qualitatively as “bright” (strong and uniform signal) or “dim” (faint signal; Fig. S3). A bright indicator line was observed in 93% of the Genobio tests and 92% of the CARBA 5 tests (Fig. 1). The small proportion of indicator lines scored as “dim” was mainly from isolates that were NDM producers (Fig. 1).

To further understand the impact of reading results beyond the recommended 15 minute time point and to inform the risk associated with these assay results, they were also recorded following 20 and 25 minute incubations. In both LFAs, background smearing and line intensity remained stable beyond the 15 minute time point (data not shown).

## DISCUSSION

Infections caused by CPOs have been increasing in Canada and globally. In this regard, from 2017 to 2021, Canada observed an increased rate of carbapenemase-producing *Enterobacterales* infections per 10,000 patient days of >150% (1). Carbapenemase detection is a key component in the efforts to curb the spread of CPOs and guide the management of CPO infections. In theory, phenotypic-based detection methods such as mCIM can be used to confirm the presence of carbapenemases of all types. However, mCIM performance has been shown to vary by carbapenemase type and does not readily provide information on the type of carbapenemase when detected. We aimed to evaluate the performance of two commercial LFAs, NG-Test CARBA 5 and Genobio K.N.I.V.O, for the detection of carbapenemases. Both assays detect five major types of carbapenemases, including KPC, NDM, VIM, OXA-48-like, and IMP. In Ontario, Canada, KPC, NDM, VIM, and OXA-48-like carbapenemases combined accounted for 93% (566/590) of carbapenemases isolated in 2022 (22).

Our study showed excellent performance of both LFAs in detecting KPC, NDM, OXA-48-like, VIM, and IMP carbapenemases in *Enterobacterales*. We observed similar performance in *P. aeruginos*a and *A. baumannii* with 100% concordance for both species. Although limited by sample size and the breadth of carbapenemase types, our findings regarding LFA performance in *P. aeruginosa* and *A. baumannii* isolates contribute to the existing literature, informing the strengths and weaknesses of LFAs for detecting carbapenemases in these organisms.

We observed some differences in the performance of these two different LFAs. Fifty of the isolates included in this study grew as mucoid colonies. Despite extended vortexing and incubation as recommended by the manufacturer, the CARBA 5 test produced an invalid result for one highly mucoid isolate. On the other hand, the Genobio assay generated a clear result with all mucoid isolates without additional processing. The Genobio test, but not the CARBA 5, exhibited weak cross-reactivity with NDM and OXA-48-like carbapenemases in a single isolate of a KPC-producing *M. morganii*. This observation has been previously described by others, including Lee et al., who investigated the relationship between inoculum size and false detection of carbapenemases by both the CARBA 5 and Genobio tests using isolates confirmed to be carbapenemase-negative by whole-genome sequencing (23). In particular, Lee et al. noted a strong propensity for false-positive NDM/OXA-48-like results among *Morganella* isolates tested by the Genobio kit. They showed that a low inoculum (one area of growth on the agar touched with a 10 µL loop) was associated with false detection of NDM and OXA-48 in 20% of carbapenemase-negative *Morganella* isolates (*n* = 10) tested, whilst a higher inoculum (half-full 10 µL loop) resulted in a 100% false positive rate (23). Neither manufacturer provides an objective, quantifiable inoculum for optimal test performance. Although it is difficult to compare our inoculum to those studied by Lee et al., our observation provides additional evidence that the Genobio LFA has a proclivity to produce false detection of NDM and OXA-48 in *Morganella* isolates. These findings underscore the need for clinical laboratories to carefully explore and monitor the effects of an inoculum size on carbapenemase detection in LFAs. In addition to issues of cross-reactivity, the Genobio test also failed to detect IMP-8 in an IMP-positive *K. pneumoniae* isolate. Similarly, Sadek et al. also observed that the Genobio failed to detect IMP-8 in three isolates (*Escherichia coli, K. pneumoniae,* and *E. cloacae* complex) that were positive for this variant (20). Additionally, they found several other IMP variants, such as IMP-2,-13, and -19, were not detected by the Genobio test but were detected by the CARBA 5 LFA (20). A previous study by Volland et al. has also shown that CARBA 5 is capable of detecting a broad range of IMP variants (17).

Time to completion and ease of interpretation are two major advantages of LFAs over many other carbapenemase detection assays. For the two LFAs evaluated herein, the time to completion was 20 minutes, with minimal hands-on time. Moreover, for both LFAs, the line indicating the presence of a carbapenemase was bright and, therefore, easily visualized in over 90% of the results. Interestingly, more than 75% of the dim test lines observed in this study were attributed to NDM. Zinc agar content, inoculum, and induction with carbapenems have been previously shown to significantly influence NDM test line intensity and detection by another LFA (24). Strong background smearing was seen in <1% of tests from both LFAs; however, the presence of “weak” background smearing was observed in substantially more tests performed with the Genobio. Although it was not encountered in this study, the combination of background smearing with a dim test line may result in falsely reporting a negative carbapenemase result, and we encourage clinical laboratories to be on alert.

Limitations with LFAs have been observed, and shifting carbapenemase prevalence along with the emergence of new types of carbapenemases will mean that clinical laboratories are likely to require complementary testing modalities to fully detect and accurately identify carbapenemases from clinical specimens. For example, an algorithm we employ involves molecular-based testing of the less commonly encountered carbapenemase types (e.g., SME, GES, and IMI) in select *Enterobacterales* isolates (e.g., *E. coli* and *K. pneumoniae*) displaying meropenem resistance and negative for carbapenemase production by LFA testing. Nonetheless, the LFAs evaluated in this study offer an efficient means to quickly detect the presence of the most prevalent carbapenemase types, thereby facilitating more timely decision-making on infection control measures and therapeutics. Overall, the findings presented here indicate very good performance of the two LFAs evaluated, with the CARBA 5 showing better all-around performance.

## SUPPLEMENTAL MATERIAL

Supplemental figures and tables spectrum.00441-25-s0001.pdf Fig. S1 to S3 and Table S1.

ASM does not own the copyrights to Supplemental Material that may be linked to, or accessed through, an article. The authors have granted ASM a non-exclusive, world-wide license to publish the Supplemental Material files. Please contact the corresponding author directly for reuse.
