# Supplementary material for: Performance evaluation of the NG-TEST CARBA 5 and Genobio K.N.I.V.O. detection K-Set lateral flow assays for the detection of carbapenemases
Source: Microbiol Spectr. 2025 Jul 18;13(8):e00441-25. doi: 10.1128/spectrum.00441-25 (PMC12323310; doi:10.1128/spectrum.00441-25)
Supplement: Supplemental figures and tables — Fig. S1 to S3 and Table S1. [file spectrum.00441-25-s0001.pdf]

# Performance Evaluation of the NG-TEST® CARBA 5 and Genobio K.N.I.V.O. Detection K-Set Lateral Flow Assays for the Detection of Carbapenemases

Chloe N. Calica, Neda Domjacic, Jennifer Dacanay, Ethel C. de Carvalho Lopes, Mariel S. Mardoquio, Yan Chen*, Ramzi Fattouh*

# Online Supplement

**Supplementary Table 1.** Overview of the 152 isolates by carbapenemase type and organism.

| Target CP[a] (Subtype) | | ***Pseudomonas*** | ***Acinetobacter*** | ***Enterobacterales*** | | | | | | | | | | |
|---|---|---|---|---|---|---|---|---|---|---|---|---|---|---|
| | | | | *Citrobacter* | *Enterobacter* | *Escherichia* | *Klebsiella* | *Kluyvera* | *Morganella* | *Proteus* | *Providencia* | *Raoultella* | *Salmonella* | *Serratia* |
| **KPC (n = 37)** | KPC-2 | | | 1 | 1 | | 4 | | 1 | 1 | | | | |
| | KPC-3 | | | | 4 | 3 | 11 | 1 | | | | 1 | | |
| | KPC-4 | | | | 1 | 1 | | | | | | | | |
| | KPC-5 | 1 | | | | | | | | | | | | |
| | KPC-6 | | | | 1 | | | | | 1 | | | | |
| | KPC UNT[b] | | | 1 | 1 | | 2 | | | | | | | |
| **NDM (n = 36)** | NDM-1 | | 4 | 1 | 1 | 6 | 11 | | 1 | | 1 | | 1 | |
| | NDM-5 | | | | | 2 | | | | | | | | |
| | NDM-6 | | | | | 1 | | | | | | | | |
| | NDM-7 | | | | | 2 | 1 | | | | | | | |
| | NDM UNT | | | 1 | 2 | 1 | | | | | | | | |
| **OXA-48-like (n = 25)** | OXA-48 | | | | 1 | | 1 | | | | | | | |
| | OXA-181 | | | | | | 5 | | | | | | | |
| | OXA-232 | | | | | | 2 | | | | | | | |
| | OXA UNT | | | 1 | 1 | 10 | 3 | | | | | 1 | | |
| **VIM (n = 13)** | VIM-1 | | | | 1 | | 2 | | | | | | | |
| | VIM-2 | 4 | | | | | | | | | | | | |
| | VIM-4 | 1 | | | | | | | | | | | | |
| | VIM-27 | | | | | | 2 | | | | | | | |
| | VIM UNT | | | | | | 3 | | | | | | | |
| **IMP (n = 6)** | IMP-1 | 2 | | | | | | | | | | | | |
| | IMP-4 | | | | 1 | | 2 | | | | | | | |
| | IMP-8 | | | | | | 1 | | | | | | | |
| **Double CP (n = 2)** | NDM-1 & OXA-232 | | | | | | 2 | | | | | | | |
| **MAIN FIVE (n = 119)** | | **8** | **4** | **5** | **15** | **26** | **52** | **1** | **2** | **2** | **1** | **2** | **1** | |
| **Other CP (n = 13)** | IMI UNT | | | | 2 | | | | | | | | | |
| | NMC-A | | | | 2 | | | | | | | | | |
| | SME-3 | | | | | | | | | | | | | 6 |
| | SME-4 | | | | | | | | | | | | | 2 |
| | SME UNT | | | | | | | | | | | | | 1 |
| **TOTAL CPO[c] (n = 132)** | | **8** | **4** | **5** | **19** | **26** | **52** | **1** | **2** | **2** | **1** | **2** | **1** | **9** |
| **NON-CPO[c] (n = 20)** | | **4** | **6** | | **4** | **2** | **4** | | | | | | | |
| **OVERALL (n = 152)** | | **12** | **10** | **5** | **23** | **28** | **56** | **1** | **2** | **2** | **1** | **2** | **1** | **9** |

[a]CP, carbapenemase; [b]UNT, untyped; [c]CPO, carbapenemase-producing organism

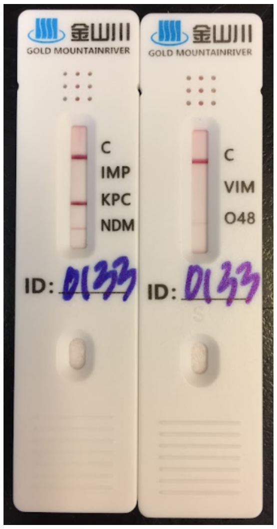


**Supplementary Figure 1.** Genobio LFA showing false positivity for NDM and OXA-48-like carbapenemase in a KPC-positive *Morganella morganii* isolate. The same observation was made upon repeat testing. This isolate was confirmed to carry only the KPC gene based on in-house single-plex PCR targeting KPC, NDM, and OXA-48-like carbapenemases.

| | None | 'Cloudy Weak' | 'Cloudy Strong' |
| --- | --- | --- | --- |
| Genobio K.N.I.V.O | 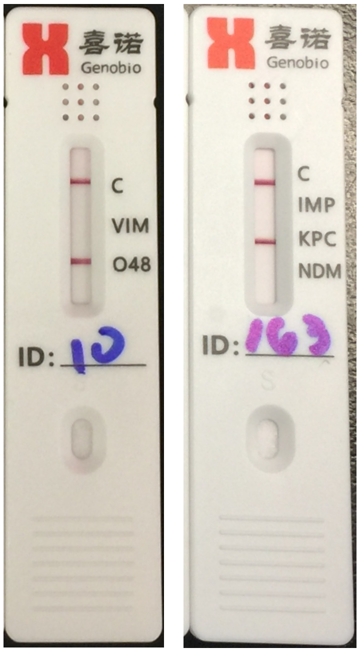 | 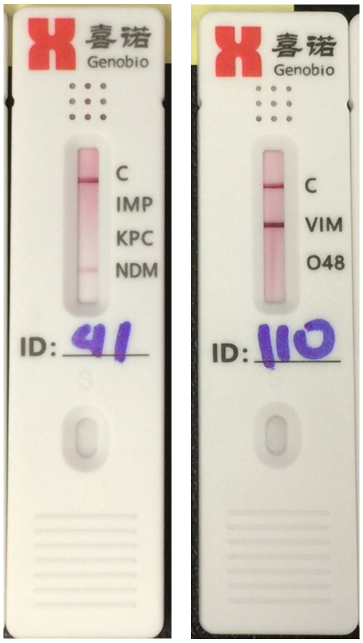 | 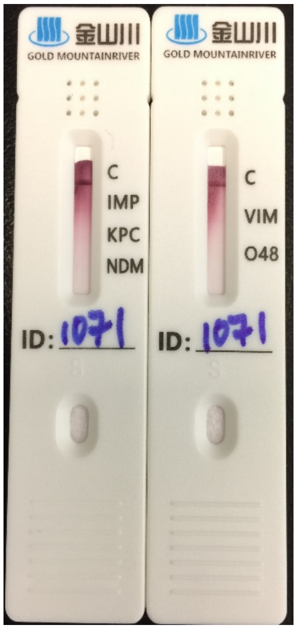 |
| NG-Test® CARBA 5 | 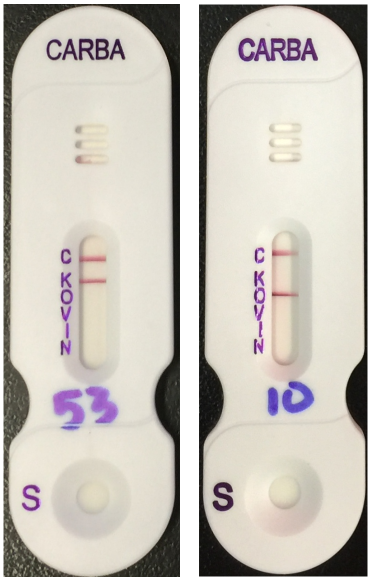 | 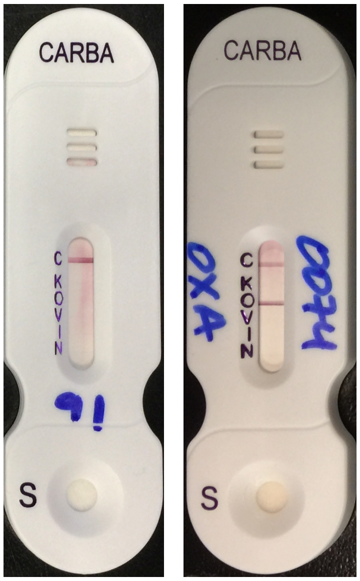 | 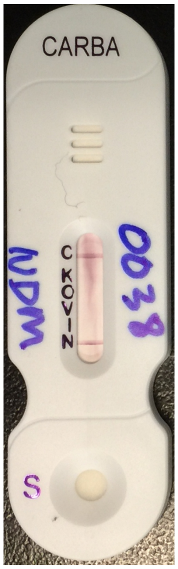 |

**Supplementary Figure 2.** Representative images of LFA cartridges showing the different background colour intensities.

<table>
<tr><th></th><th>‘Dim’</th><th>‘Bright’</th></tr>
<tr><td>Genobio K.N.I.V.O</td><td>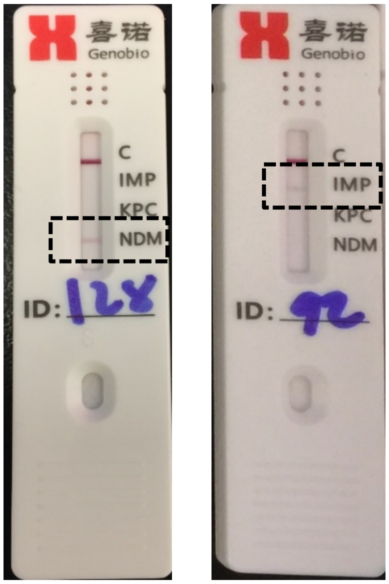
</td><td>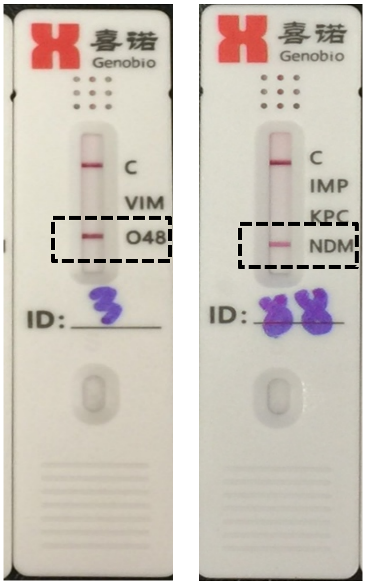
</td></tr>
<tr><td>NG-Test® CARBA 5</td><td>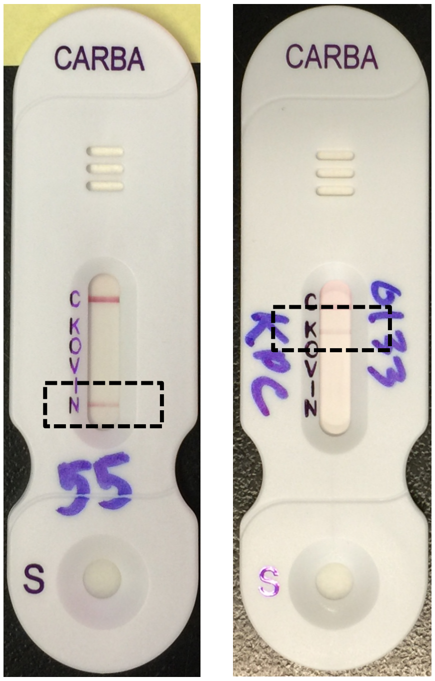
</td><td>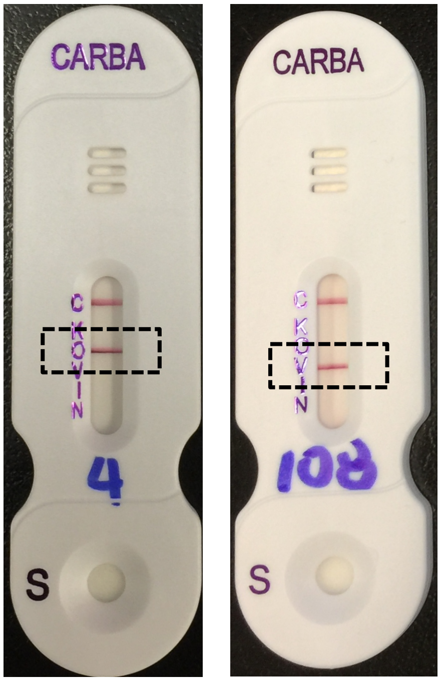
</td></tr>
</table>

**Supplementary Figure 3.** Representative images of LFA cartridges showing the different line intensities. Each cartridge was ranked based only on the intensity of the positive carbapenemase-type indicator line and not the control line. These lines are indicated by a black dashed rectangle in the image of each cartridge.
